# Insights on Stability Constants and Structures of Complexes between Coumarin Derivatives and Pb(II) in Aqueous Media

**DOI:** 10.3390/molecules29091911

**Published:** 2024-04-23

**Authors:** Emilia Furia, Vincenzo Lettera, Anna Napoli, Donatella Aiello

**Affiliations:** Department of Chemistry and Chemical Technologies, University of Calabria, 87036 Rende, Italy; emilia.furia@unical.it (E.F.); vincenzo.lettera@unical.it (V.L.); amc.napoli@unical.it (A.N.)

**Keywords:** Lead(II) coumarin complexes, stability constants, complexation sites, LD-MS characterization

## Abstract

In the frame of a systematic study on the sequestering ability of natural antioxidants towards metal cations, here the complexation of coumarin-3-carboxilic acid (HCCA) with Pb(II) and the overall stability constants of the resulting complexes, at 37 °C and in 0.16 M NaClO_4_, are discussed. Reaction of Pb(ClO_4_)_2_ with HCCA in an aqueous medium at a pH range from 2 to 6 and various ratios (1:1–1:10) yielded the Pb–CCA complexes, which were characterized spectrometrically by laser desorption ionization mass spectrometry (LD-MS). LD-MS has provided the composition and structure of Pb–CCA species according to the speciation model proposed on the basis of the potentiometric data. The graphic representation of the complex’s concentration curves is given by the distribution diagram, which provides a whole depiction of the species present in the solution at the selected pH ranges.

## 1. Introduction

The poisoning effects caused by toxic metal ions represent one of the most important health and social issues in industrialized and emerging countries. For this reason, they have become an important object of study for the scientific community, which continuously searches for methods and therapies to determine and regulate the presence of these metal ions in humans and the environment. Among them, lead, whose toxicity has been reviewed extensively [1,2,3,4,5], is a harmful metal ion causing adverse environmental and health problems. Its toxic effects arise from its multipotent involvement in interactions with enzymes and nucleic acids, where inhibition of biochemical pathways often constitutes the source of symptomatic physiological aberrations. The borderline “soft–hard” character of lead was responsible for the metal ion’s interactions with biomolecules; furthermore, according to its nature, this metal ion may fit in the binding sites of several biomolecules and adapt to different coordination geometries [6]. As reported in the literature, Pb(II) is able to react with mono-/poly-carboxylic acids, yielding complexes with a multitude of ligand coordination modes and high coordination numbers [7,8,9,10]. Research on chelating agents, capable of reducing the concentration of this toxic cation, is currently insufficient, as it should be based primarily on biological control methods and not on chemicals. Chelating agents for specific metal ions are not necessarily the best and most common drug. Specificity, the stability of the complex formed, and knowledge about treatment duration, temporary or lifelong, are fundamental to the choice of chelating agent. According to the number of coordination groups on the molecule that can simultaneously bind the target metal ion, chelating agents can be divided into bidentate, tridentate, and polydentate.

Among oxygen-type ligands, coumarins and their derivatives have stimulated interesting research due to their several properties [11,12,13,14]. Coumarins belong to a family of natural and synthetic compounds containing a heterocycle of fused benzene and α-pyrone rings as their core structure. Their biological properties are relevant in medicine and in the food and chemical industries [15,16,17] because of antioxidant activities along with anti-inflammatory action and enzyme interactions [18,19]. However, they have not been thoroughly evaluated for their ability to detoxify metals that are toxic to organisms and recalcitrant to the environment. Their antioxidant capacity allows them to alleviate oxidative stress by scavenging of free radicals and chelating metal ions. This last aspect is relevant in clinical settings because accumulation of metal ions in the body at concentrations higher than the optimum level can lead to metal toxicity. This is caused by metal-induced formation of reactive oxygen species (ROS) and reactive nitrogen species (RNS), resulting in (per)oxidation of biological molecules.

Coumarin-derived complexes can be obtained through different coordination modes with varying spectroscopic properties and have been extensively studied for their great therapeutic potential due to the wide spectrum of physicochemical properties and biological activities, which can be enhanced by combining coumarin moiety with metal ions [20].

**Figure 1:**
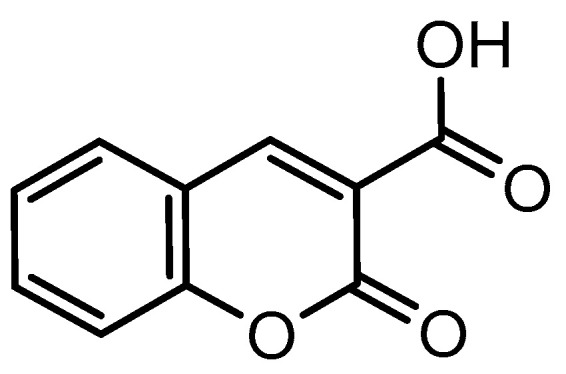
Chemical structure of HCCA.

Previously, the biological action of coumarin-3-carboxylic acid (HCCA, Figure 1) has been potentiated through complexing with metals [21].

This ligand can act as a monodentate via carboxylic moiety involving one or both oxygen atoms, as well as a bidentate ligand through the lactone and the carboxylic oxygen to realize a high stabilized 6-membered metallocycle [22].

The complexation ability of coumarin-3-carboxylic acid towards selected metal cations in aqueous solutions at 37 °C and in 0.16 mol L^−1^ [i.e., inert salt NaCl or NaClO_4_ depending on specific metal ions] has been reported [22,23,24,25]. In these previous investigations it has been verified that, with the single exception of Fe(III), HCCA exhibits only one mode of binding, in which the deprotonated carboxylic group acts as a bidentate chelating ligand.

The present study aimed to investigate the formation of Pb-CCA complexes in 0.16 M NaClO_4_ at 37 °C to determine their stoichiometry and corresponding stability constants.

Taking into account the importance of the ligand and metal ion for the human body, these experimental conditions were chosen to reproduce the ionic strength and temperature of biological fluids. Potentiometric measurements and laser desorption mass spectrometry (LD-MS) were utilized to determine the speciation profile and coordination mode, respectively. The paper will discuss the equilibrium behaviour and speciation model of the complexes in an aqueous solution at various pH ranges, as well as their possible structures.

## 2. Results and Discussion

### 2.1. Potentiometric Measurements

Potentiometric measurements were performed on the Pb(II)–HCCA system to obtain the stoichiometry of the complexes and the magnitude of the corresponding stability constants to depict the speciation profiles. The complex formation equilibria were studied at 37 °C and in 0.16 M NaClO_4_. This background salt was chosen to control the ionic strength due to its high inertia towards complexation [26]. Results are provided according to the general equilibrium reported in Equation (1), which includes all the possible complexes formed in solution:

pPb^2+^ + qOH^−^ + rCCA^−^ ⇄ Pb_p_(OH)_q_(CCA)_r_^(2p–q−r)^   log β_pqr_ (1)

the most probable p, q, and r stoichiometric coefficients and the corresponding stability constants log β_pqr_ were achieved by a numerical procedure [27], and the obtained values are reported in the Table 1.

The constants of the main cationic hydrolysis products [28] (i.e., Pb(OH)^+^, Pb_4_(OH)_4_^4+^, Pb_3_(OH)_4_^2+^, and Pb_6_(OH)_8_^4+^) and the acidic constant of the ligand [20] were kept invariant in the numerical treatment to determine stability constants. The experimental data (C_M_, C_L_, C_A_, C_B_, [H^+^]) were firstly processed by graphical procedures, which consist in a comparison of experimental plots with model functions [29]. To explain the experimental data, the simple hypothesis was made that the main reaction products are binary complexes and mononuclear in metal ion, i.e., *q* = 0 and *p* = 1 in Equation (1), respectively, formed according to equilibrium (2):

Pb^2+^ + *r* CCA^−^ ⇄ Pb(CCA)*r*^(2−r)^   log β_10r_ (2)

**Figure 2:**
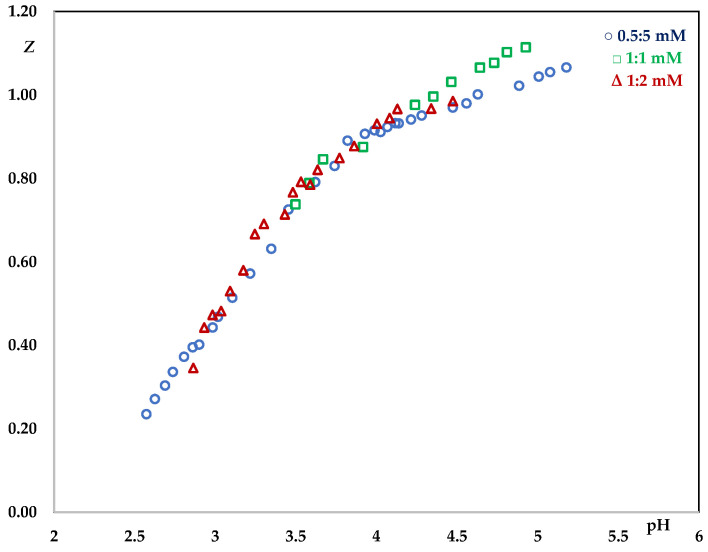
Z as a function of log([HCCA]/[H_3_O^+^]).

The validity of this assumption was tested by constructing the graph Z, which represents the average number of ligands for metal ion (Equation (3)), as a function of log ([HCCA]/[H_3_O^+^]) from the primary data (Figure 2).

Z = (C_A_ − [HCCA] − [CCA^−^])/C_B_ (3)

Thus, when complexes of the general formula Pb(CCA)*_r_*^(2−*r*)^ predominate, the points Z versus log ([HCCA]/[H_3_O^+^]), at different C_L_ and C_M_, should fall on a unique curve.

Most of the experimental points, registered at three different metal/ligand ratios, fall on a unique curve which tends to 1, confirming that the coordination of ligand to metal ion is accompanied by the loss of a single proton. However, small but systematic deviations from the model including just one simple species were observed, confirming that some additional complex was present. The probable composition of the complexes, responsible for these deviations, was achieved by numerical treatment of the data set [27]. This analysis allows us to determine the stability constants of species in solution based on the potentiometric titration data, and to minimize the error-square sum based on measured electrode potentials. There exists a model of the equilibrium system that adequately accounts for the experimental observations, which is specified by a set of coefficients, one for each species formed, and all least-squares refinements are performed in terms of an assumed model. Examination of a series of models ought to generate that which is closest to the true behaviour. The process of selecting the optimal model is referred to as species selection.

**Figure 3:**
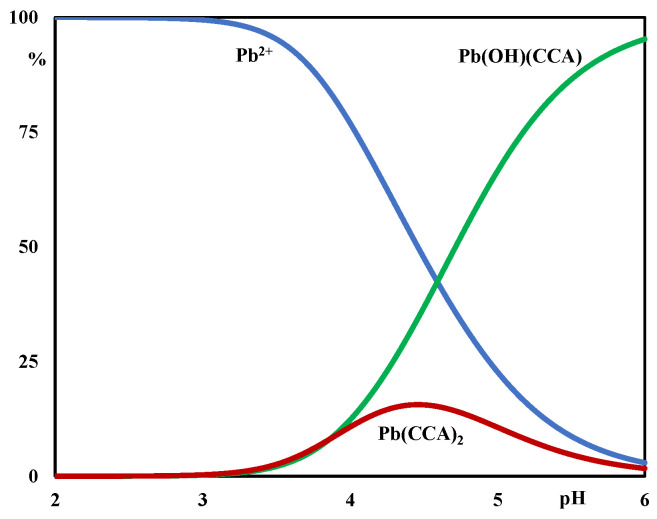
Distribution diagram in the presence of HCCA. C_M_ = 1.0 mM and C_L_ = 5.0 mM.

Firstly, we attempted to explain the experimental data using a single complex. We found that the best minimum was achieved with the mixed mononuclear species, Pb(OH)(CCA), and the square error sum U was reduced by also considering the Pb(CCA)_2_ complex. No other species, added to enhance the fit, were retained and therefore excluded from the system. The distribution diagram reported in Figure 3 displays that all the complexes reached significant percentages and started to form in the acidic pH range.

It is evident from Figure 3 that none of the metal hydrolytic species reached significant percentages.

### 2.2. Mass Spectrometry Analysis

**Figure 4:**
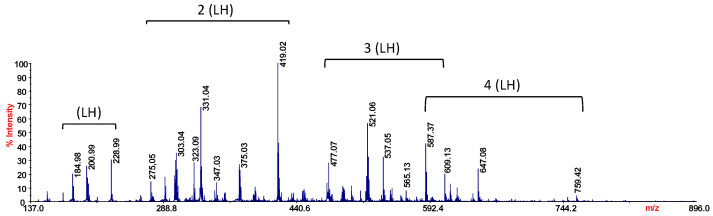
LD-MS of HCCA.

Mass spectrometry is an instrumental technique frequently adopted to clarify structures and coordination sites in complexes in which metals can be chelated by ligands [30,31]. Ionization is a very complex phenomenon, since it could comprise the formation of protonated, deprotonated, cationized, and sometimes even radical species, affected by the synergic effect of the matrix, solvent composition, solution pH, and acid–base properties of the analytes [32]. Therefore, even if the study of metal–organic ligand complexes using MALDI-TOF MS is an exciting, diverse, lively, and rather interesting area, the choice of the matrix, the pH conditions, and the nature of the ligand deeply affect the success of the analysis. The presence of the matrix protects the analytes and improves desorption/ionization phenomena, as well as simplifying sample preparation. Furthermore, without a matrix, large amounts of fragmentation could be observed, which in some cases is useful for understanding the ionization behaviour of molecules, while in other cases it is a complication for the interpretation of mass spectrometry data [33]. Metal ion–matrix complexes can be considered as pre-formed ions and it was well established that MALDI mass spectra qualitatively reflect the quantity of preformed complexes in the solid target [34,35]. However, only the complexes surviving the ionization processes can be detected and analyzed with the mass spectrometer. Different grades of adduct generation can be observed, depending on the amount of energy transfer to the analyte, which is usually controlled by the matrix. The distribution of the analyte and matrix adducts proves the presence of the multi-ion pairs in crystals on the MALDI plate and that ionization is essentially accomplished by charge separation processes [36]. In particular, the structures of the complexes of lead ion and coumarin-3-carboxylic acid (HCCA) were investigated by High Resolution (HR) Laser Desorption Ionization (LD) Mass Spectrometry (MS). The use of HCCA as a ligand falls within the specific case where the ligands also act as a matrix to promote the formation of the ions in the source. Indeed, the ligand exhibits a highly conjugated double-bond structure typically observed in the organic acids regularly used as a MALDI matrix. Literature data report the use of coumarins as a matrix for MALDI-MS analysis of DNA [37] and hydrophobic compounds, as steroids and sterols, by MALDI-FT ICR MS in positive-ion mode [38]. Therefore, it was interesting to explore the ionization efficiency and behaviour of the ligand with the aim of studying its chelating capabilities in more detail. Studying the analyte alone offers several advantages: the crystal is homogeneous, spectra are simpler, and all signals can be ascribable to the analyte. Moreover, when the analyte is a ligand, the characterization of the interaction with a metal ion becomes more reliable. HCCA is expected to have a higher affinity for hydrophilic compounds and to suppress dissociation of labile regions; therefore, its behaviour in the gas phase was initially checked in LD-MS conditions [39]. Coumarin-3-carboxylic acid is expected to form self-associated homodimers through π-stacking interactions [40]; indeed, in Figure 4 it is possible to note the presence of ions, proving information on several protonated and cationized species. The spectrum shows the formation of four cluster regions ascribable to from one to four units of HCCA, as shown in Table 2.

The comparison of the measured experimental isotopic distribution with the theoretically calculated distribution of the expected summary formula suggests that the ion clusters of *m*/*z* 228.99 ([C_10_H_6_O_4_K]^+^, [LH+K]^+^), 419.02 ([C_20_H_12_O_8_K]^+^, [2LH+K]^+^), and 609.05 ([C_30_H_18_O_12_K]^+^, [3LH+K]^+^) correspond to one, two and three HCCA units, respectively, while the ion cluster of *m*/*z* 755.08 arises from the loss of 44 Da (CO_2_) from four HCCA units ([C_39_H_24_O_4_K]^+^, [4LH-CO_2_+K]^+^). Several of the observed ion species arise from the loss of 44 Da (CO_2_), leading to the formation of the ion species reported in Table 2. It is interesting to note that the clusters of coumarin-3-carboxylic acid show an evident stability in the adopted experimental conditions, confirming the predicted affinity for hydrophilic compounds.

In order to evaluate the behaviour of lead cation in the gas phase, the metal was also studied without the ligand. Literature data report interesting examinations regarding the gas phase ion chemistry of metal clusters [41,42]. This is mainly due to different aspects; among others, cluster exploration could be the ideal interface between experimental and theoretical research [43]. By studying the gas phase behaviour of metals it is possible to collect promising information useful to understand the chemistry of metal nanoparticles, and in this perspective the mass spectrometers are ideal reactors to study size-selected “pure” metal clusters as well as metal compounds [44,45,46]. In particular, transition metals have been the subject of intense research to evaluate their bonding, growth patterns, and the possibility of applications in nano-technology [47]. The electronic and electric properties of lead clusters have been studied experimentally and theoretically, including their excitation by strong laser pulses and their mass spectrometric fragmentation [48]. Schäfer et al. [49] reported the formation of a plasma plume by irradiating a lead rod with the focused light of an Nd:YAG (yttrium aluminium garnet) laser. The plume, cooled down in a flow of helium gas and condensed, was able to form clusters (Pb_n_). In the adopted experimental conditions, 1 μL of a solution containing Pb(ClO_4_)_2_ was loaded on the plate and dried at room temperature, and the LD-MS spectra were directly acquired by irradiating the crystal with the Nd:YAG laser.

**Figure 5:**
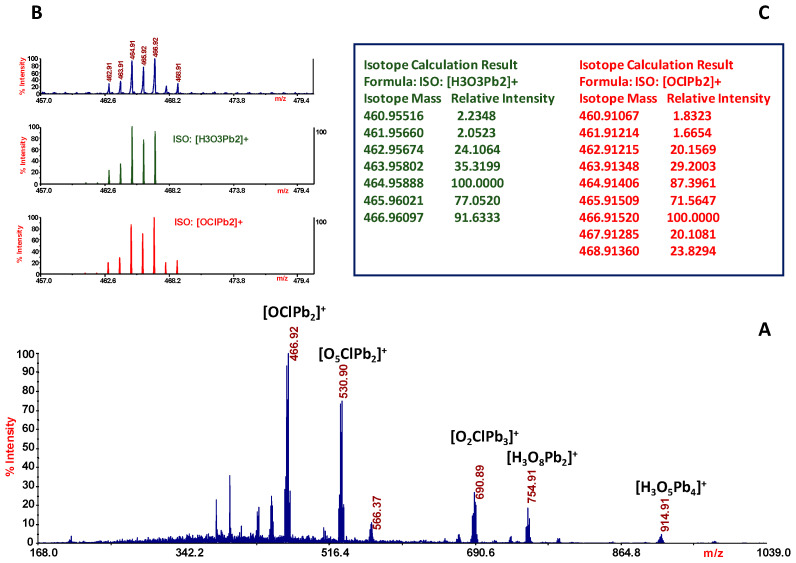
(**A**) LD-MS spectrum of Pb(ClO_4_)_2_ solution. Inset (**B**): spectra show expanded experimental isotopic mass distribution and theoretical isotopic mass distribution of ionic species of *m*/*z* 466.92; inset (**C**): lists isotope calculation results.

Figure 5 shows several species in the LD(+) MS of the Pb(II) system, which can be identified by the distribution and intensity of the signals. The spectrum (Figure 5A) shows the formation of five more intense signals. To assign the formula, each peak was evaluated by comparing the measured experimental isotope distribution with the theoretically calculated distribution of the expected summary formula. Figure 5B,C show the study performed around the ion peak of *m*/*z* 466.92. The ion calculation results suggested two possible formulas, ISO: [H_3_O_3_Pb_2_]^+^ and ISO: [OClPb_2_]^+^, through elemental analysis. The assignment can be only made by considering the arrangement of the isotope masses and the relative intensities. Comparison of the measured experimental isotopic distribution with the theoretically calculated distribution of the expected summary formula suggests that the ion cluster of *m*/*z* 466.92 can be assigned to [OClPb_2_]^+^. The same procedure was followed for all observed signals and all chemical formulas are reported in Table 3.

**Figure 6:**
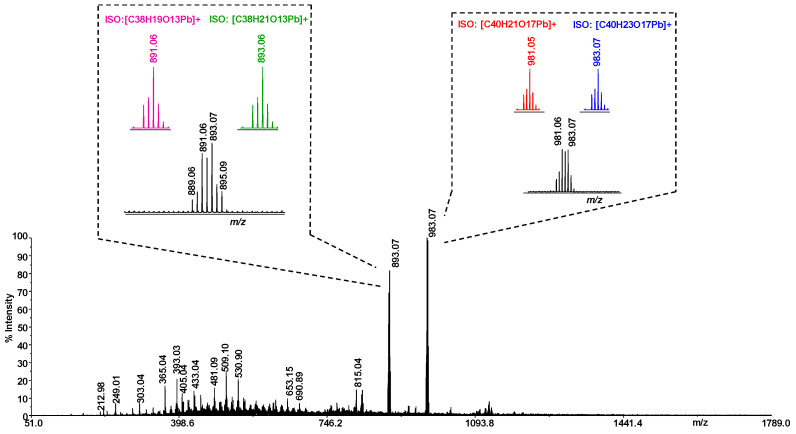
LD-MS spectrum of Pb–HCCA system. Inset spectra show expanded experimental isotopic mass distribution and theoretical isotopic mass distribution of ionic species of *m*/*z* 893.07 and 981.06.

The structure of the complexes of Pb(II) with coumarin-3-carboxylic acid were further investigated by high-resolution (HR) laser desorption (LD) MS and MS/MS experiments [31,32]. Signals corresponding to complex metal: ligand with 1:4 stoichiometry are the most intense signals in the spectrum for the investigated systems (Figure 6). The molecular masses derived from these measurements are in good agreement with the calculated masses, within 5 ppm (Table 4).

The LD(+) MS spectrum of the Pb–HCCA system (Figure 6) shows the formation of two more intense signals. To assign the formula, each peak was evaluated by comparing the measured experimental isotope distribution with the theoretically calculated distribution of the expected summary formula. In Figure 6, insets show the study performed around the ion peaks of *m*/*z* 983.07 and 893.07. The ion calculation results suggested two possible formulas ([C_40_H_23_O_17_Pb]^+^, *m*/*z* 983.07; [C_40_H_21_O_17_Pb]^+^, *m*/*z* 981.05) for the ion of *m*/*z* 983.07. It is therefore possible to propose that this species results from the overlapping of two isotope cluster ions of two different species, both with stoichiometry 1:4 (metal: ligand), as shown in Table 4. Nevertheless, the stabilization of complexes with such a high number of ligands may in part be due to π-stacking interactions between the ligand molecules. A similar study was conducted on the ion of *m*/*z* 893.07, whose signal pattern appears to be due to the overlapping of two different ion species (*m*/*z* 893.07, [C_38_H_21_O_13_Pb]^+^; *m*/*z* 891.06 [C_38_H_19_O_13_Pb]^+^).

**Figure 7:**
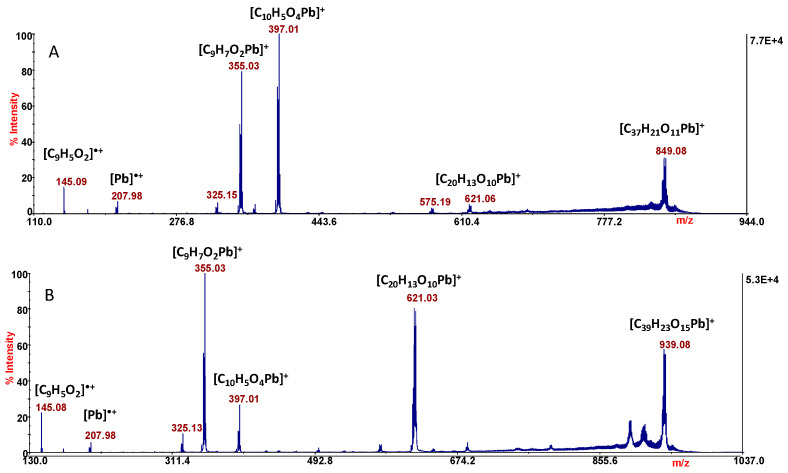
MS/MS spectra of the ions of *m*/*z* 893.07 (**A**) and 983.07 (**B**).

MS/MS experiments were performed in order to assign the sites of metal coordination and clarify the structures of the complexes. The fragmentation patterns of the ions of *m*/*z* 983 and 893 in MS/MS analysis are similar to each other. In Figure 7 are reported the MS/MS spectra of both ion pairs.

The product ion of *m*/*z* 849 (Figure 7A) is formed by the neutral loss of 44 Da (CO_2_) from the precursor, and the ion of *m*/*z* 621 is ascribable to the loss of two ligand units. The most informative peaks are those of *m*/*z* 397 and 355; in both the stoichiometry is 1:1, Pb: ligand. In particular, the ion of *m*/*z* 397.01 ([C_10_H_5_O_4_Pb]^+^) is ascribable to the species [PbCCA]^+^, in which the lead ion is linked to the coumaric acid through the carboxylic group with the assistance of the lone pair of the pyranone oxygen.

The coordination is stabilized by the formation of a six-membered cycle, which is possible only when the carboxylate and lactone moieties are involved in the metal coordination. This is also confirmed by the neutral loss of CO_2_ (−44 Da) leading to the formation of the contiguous ion of *m*/*z* 355. However, all the observed signals are ascribable to ions containing lead cation, the only exception being the radical ion of *m*/*z* 145, which can be only assigned to the ligand ([C_9_H_5_O_2_]^•+^). Of particular interest is the formation of the ionic species of *m*/*z* 207.98, which is the radical cation of the metal alone. The release of “free metal” ([M]^•+^) in the experimental adopted condition is a common phenomenon and becomes a suitable strategy to identify different metal species in environmental samples, by LD MS/MS analysis [50]. The MS/MS spectrum of the ion of *m*/*z* 983 provides the same information, with the only difference between the two MS/MS spectra (Figure 7A,B) being related to the appearance/intensity of the product ions.

## 3. Materials and Methods

### 3.1. Materials and Sample Preparation

All solutions were freshly prepared with bidistilled water, freed from any organic impurities by means of a Milli-Q system (Millipore, Burlington, MA, USA). Lead(II) perchlorate, perchloric acid, sodium perchlorate stock solutions, and the sodium hydroxide titrant solutions were prepared and standardized as previously described [23,24,27]. HCCA (Sigma, St. Louis, MO, USA, ≥99%) was kept in a desiccator over silica gel and was used without further purification. Considering the low solubility of the ligand in water, all the experiments were carried out by adding an exactly known and weighed quantity of solid HCCA in the titration’s apparatus. After addition of the other reagents, the pH of test solutions was stepwise increased by adding NaOH standard solutions. When the complexation equilibria take place, HCCA dissolved into the aqueous medium to form complexes with metal cation.

### 3.2. Potentiometric Measurements

Complexation equilibria were studied in 0.16 M NaClO_4_ at 37 °C, by measuring competition for H^+^ between ligand and metal cation. The acidic constants of the ligand were taken from the literature [23]. Cell arrangement and electrodes were previously described [27]. The measurements were performed as potentiometric titrations with cell (G):

Reference Electrode/Test Solution/Glass Electrode   (G)(4)

All titrations were conducted with a programmable computer-controlled data acquisition switch unit 34,970 A from Hewlett and Packard. The EMF values were measured at a precision of 10^−5^ V using an OPA 111 low-noise precision DIFET operational amplifier.

The general composition of the test solution was *C*_M_ M Pb(ClO_4_)_2_, *C*_L_ M HCCA, *C*_A_ M HClO_4_, *C*_B_ M NaOH, and (0.16-2*C*_M_-*C*_A_-*C*_B_) M NaClO_4_. Metal and ligand concentrations ranged from (0.5 × 10^−3^) to (5.0 × 10^−3^) M and the ligand-to-metal ratio was varied between 1 and 10. The pH ranged from 2.0 to 6.0, when the precipitation of a neutral species takes place, and it was dependent on the specific ligand-to-metal ratio investigated. After the addition of the reagents, the glass electrode by Metrohm acquired a constant potential within 60 min which remained unchanged within 0.1 mV. The EMF of cell (G) can be written, in mV, at the temperature of 37 °C, as Equation (5):

*E* = *E*° + 61.54 log [H^+^] + *E_j_* (5)

where *E*° is the constant in each series of measurements and the value of *E_j_* was taken from the literature [23]. In the first part, *E*° was determined for each titration in the absence of ligand and metal. In particular, the acidity of the test solution (i.e., 20 mL 0.16 M NaClO_4_) was varied by adding 2.5 mL of 10 mM HClO_4_ titrant solution. In the [H^+^] range 10^−4^–10^−2^, M constant values in the range from 310 to 350 mV, to within 0.1 mV, were calculated. In the second part, the acidity was decreased stepwise by adding NaOH standard solution using a manual burette. The titrant’s concentration was varied from 5.0 to 25.0 mM and the final titrant volume from 10 to 50 mL, depending on the specific metal-to-ligand ratio investigated. A continuous slight flow of nitrogen gas was passed through the test solutions, stirred magnetically during titrations, to avoid oxygen interference. The cell assembly was placed in a thermostat kept at (37.0 ± 0.1) °C.

### 3.3. Mass Spectrometry Analysis

The free metal and ligand were first analyzed by LD-MS experiments (5800 AB SCIEX, Darmstadt, Germany), in order to evaluate ionization behaviour. 1 mg of HCCA powder was solubilized in 2 mL of H_2_O/CH_3_CN (4:6, *v*:*v*) and 1 μL of the solution was directly analyzed by LD-MS. Similarly, 1mg of Pb(ClO_4_)_2_ was solubilized in H_2_O/CH_3_OH (1:1, *v*:*v*) and then analyzed by mass spectrometry. The Pb–HCCA complexes were prepared starting from 2mmol of ligand dissolved in a solution of H_2_O/CH_3_CN (4:6, *v*:*v*), while a solution of one equivalent of Pb(II) was added over stirring. Complexes were not isolated and 1 μL of the resulting reaction mixture was directly loaded on MALDI plate and analyzed by positive-ion-mode mass spectrometry. LD-MS analyses were performed using a 5800 MALDI TOF–TOF Analyzer (AB SCIEX, Darmstadt, Germany) equipped with a neodymium–yttrium–aluminium–garnet laser (laser wavelength 349 nm). At least 3500 laser shots were typically accumulated with a laser pulse rate of 400 Hz in the MS mode with a mass accuracy of 5 ppm. Each sample (free metal, free ligand, and reaction mixture) was sampled in triplicate, and for each spot were acquired at least three spectra, for a total of nine data points for each sample. All data presented in this work are averages of three replicates. Calibration of the instrument was performed according to the instructions of the manufacturer to optimize mass assignment, calibration, resolution and sensitivity. MS/MS experiments were performed at a collision energy of 1 kV. Spectra were acquired accumulating up to 4000 laser shots and were recorded in positive mode. 

All spectra were handled using Data Explorer version 4.0; in particular, for each signal was performed the comparison of the measured experimental isotopic distribution with the theoretically calculated distribution of the expected summary formula.

## 4. Conclusions

The speciation model and the formation constants of the Pb(II)–HCCA system were proposed on the basis of potentiometric results. Mass spectrometry was a potent tool to explore the ionization efficiency and behaviour of the ligand with the aim of studying its chelating capabilities in more detail. In particular, the ability of the coumarin-3-carboxylic acid to form self-associated homodimers through π-stacking interactions was reported. The LD-MS analysis was also adopted to evaluate the behaviour of lead cation in the gas phase, studying the metal with and without the ligand. Metal–ligand complexes were studied in order to provided information regarding the stoichiometry of the complexes and the chelation site was clarified by MS/MS investigation. The most informative fragments suggest that the metal ion is linked to the coumaric acid through the carboxylic group with the assistance of the lone pair of the pyranone oxygen. The coordination is stabilized by the formation of a six-membered cycle, which is possible only when the carboxylate and lactone moieties are both involved in the metal coordination.

## Figures and Tables

**Table 1:** Formation of complexes of HCCA with Pb(II) according to general Equation (1). Values of log β_pqr_ in NaClO_4_ 0.16 M at 37 °C were obtained by numerical procedure (standard deviations are reported as 3 σ).

(*p*,*q*,*r*)	Complexes	log β*_pqr_* ± 3σ
(1,1,1)	Pb(OH)(CCA)	10.77 ± 0.01
(1,0,2)	Pb(CCA)_2_	3.6 ± 0.2

**Table 2:** MS ions of HCCA (LH).

Species	*m*/*z*	Error (ppm)	Chemical Formula
[LH+K]^+^	228.99	3.5	[C_10_H_6_O_4_K]^+^
	200.99	3.6	[C_9_H_6_O_3_K]^+^
[LH-CO_2_+K]^+^	184.98	3.1	[C_9_H_6_O_2_K]^+^
[2LH+K]^+^	419.02	3.1	[C_20_H_12_O_8_K]^+^
[2LH-CO_2_+K]^+^	375.03	3.0	[C_19_H_12_O_6_K]^+^
	347.03	3.5	[C_18_H_12_O_5_K]^+^
[2LH-2CO_2_+K]^+^	331.04	3.9	[C_18_H_12_O_4_K]^+^
	323.09	4.0	[C_19_H_15_O_5_]^+^
	303.04	3.8	[C_17_H_12_O_3_K]^+^
	275.05	3.5	[C_16_H_12_O_2_K]^+^
[3LH+K]^+^	609.05	5.0	[C_30_H_18_O_12_K]^+^
[3LH-CO_2_+K]^+^	565.05	3.1	[C_29_H_18_O_10_K]^+^
	537.05	3.4	[C_28_H_18_O_9_K]^+^
[3LH-2CO_2_+K]^+^	521.06	3.5	[C_28_H_18_O_8_K]^+^
[3LH-3CO_2_+K]^+^	477.07	3.5	[C_27_H_18_O_6_K]^+^
[4LH-CO_2_+K]^+^	755.08	3.9	[C_39_H_24_O_14_K]^+^
	647.12	3.6	[C_36_H_23_O_12_]^+^
	587.13	3.1	[C_35_H_23_O_9_]^+^

**Table 3:** MS ions of PbClO_4_.

*m*/*z*	Error (ppm)	Chemical Formula
466.92	3.0	[OClPb_2_]^+^
530.90	3.1	[O_5_ClPb_2_]^+^
690.89	3.4	[O_2_ClPb_3_]^+^
754.91	3.0	[H_3_O_8_Pb_3_]^+^
914.91	3.5	[H_3_O_5_Pb_4_]^+^

**Table 4:** MS and MS/MS ions of Pb–HCCA system.

*m*/*z*	Error (ppm)	Chemical Formula
983.08	3.5	[C_40_H_23_O_17_Pb]^+^
981.06	3.7	[C_40_H_21_O_17_Pb]^+^
893.07	3.7	[C_38_H_21_O_13_Pb]^+^
891.06	3.8	[C_38_H_19_O_13_Pb]^+^
**MS/MS Fragments**		
***m*/*z***	**Error (ppm)**	**Chemical Formula**
939.08	4.1	[C_39_H_23_O_15_Pb]^+^
849.08	3.9	[C_37_H_21_O_11_Pb]^+^
621.03	3.8	[C_20_H_13_O_10_Pb]^+^
397.01	4.0	[C_10_H_5_O_4_Pb]^+^
355.03	4.2	[C_9_H_7_O_2_Pb]^+^
207.98	3.7	[Pb]^•+^
145.03	4.0	[C_9_H_5_O_2_]^•+^

## Data Availability

Data are contained within the article.
